# Phase-Oriented Therapy for Dissociative Identity Disorder: A Narrative Review

**DOI:** 10.1192/bjo.2026.11079

**Published:** 2026-06-30

**Authors:** Emerald Cheung, Nyan Lin Khant

**Affiliations:** University of Buckingham, Buckingham, United Kingdom

## Abstract

**Aims:**

Dissociative Identity Disorder (DID), previously known as Multiple Personality Disorder, is characterised by the presence of two or more distinct personality states, often arising as a coping mechanism for severe and prolonged trauma. Based on the structural dissociation theory, Phase-Oriented Therapy is currently the treatment model recommended by the International Society for the Study of Trauma and Dissociation. It comprises three phases: Phase 1 (Stabilisation), Phase 2 (Trauma Processing) and Phase 3 (Integration and Rehabilitation). Despite its clinical significance, the evidence base remains limited, with phase-specific strategies underexplored. This review examines how dissociative parts are managed throughout the three phases of Phase-Oriented Therapy in individuals with DID, emphasising therapeutic goals, key techniques used, clinical challenges and treatment outcomes.

**Methods:**

A narrative review was conducted using the SPIDER framework. Literature was sourced from databases such as PubMed, Wiley, EBSCO and Google Scholar using terms related to DID and Phase-Oriented Therapy. Included studies consisted of case reports, observational studies, systematic reviews and clinical guidelines.

**Results:**

Phase 1, the stabilisation phase, was consistently identified as foundational, with therapeutic aims including safety, emotion regulation and psychoeducation. Reported outcomes included improved affect regulation and self-control. However, many patients struggled to progress beyond this phase due to a significant level of symptoms or early dropout.

Phase 2 focused on trauma processing through exposure-based and cognitive restructuring techniques, including inner communication and grief processing. Outcomes included greater affect tolerance, reduced self-blame and improved internal communication between parts. However, several studies noted delayed symptom improvement, with treatment effects often emerging only during follow-up.

Phase 3 emphasised self-integration, functional adaptation and acceptance of functional multiplicity, defined as cooperative coexistence of parts without complete fusion. Patients showed improvements in daily functioning and a renewed sense of purpose, though some residual dissociative symptoms remained at the end of treatment.

Across studies, common challenges included high attrition rates, underdiagnosis and limited access to therapists trained in dissociation-specific approaches. Variations in treatment length,intensity and manualisation also contributed to inconsistent outcomes.

**Conclusion:**

Phase-Oriented Therapy offers a structured, trauma-informed and clinically meaningful approach to managing DID. While current evidence highlights its clinical value, significant gaps remain, particularly around phase transition criteria, long-term outcomes and treatment standardisation. Future studies should prioritise controlled trials, longitudinal follow-up and more precise definitions of phase progression to strengthen the empirical foundation for this treatment model.

